# Diseases of the Blood

**Published:** 1906-08-11

**Authors:** 


### DISEASES OF THE BLOOD.

**Arterio-sclerosis.**—Among the causes which lead to this condition are the infective processes, such as typhoid, septicæmia, syphilis, and lead-poisoning. Barr, Christophers, and others think that the bacillus coli has a similar power, and show that the blood of persons who suffer from the disease much more often agglutinates the bacillus coli than the blood of normal persons does. In cases examined by John Hay, the percentage of persons thus affected whose blood caused clumping was 53, as compared to 20 in normal individuals. Alcohol does not seem to have much direct effect, but Barr [1] claims that continued high tension and an excess of nitrogenous food are powerful factors. In connection with this point he accepts the recent results of Chittenden as to the small amount actually needed, but it has been pointed out elsewhere that these results need confirmation, and that it is totally unknown how individuals on such a restricted diet stand a sudden attack of disease or even very heavy labour. It is, indeed, noticeable that such persons find a great difficulty in increasing their food if it is desirable to do so. Barr, indeed, confesses that a great reduction of the purin bodies in food may sometimes be harmful, for, as Oliver has pointed out, these substances act during digestion as distributors of lymph to the tissues, an office which the nutritious elements of food themselves are unable to carry out. Still, in the treatment of arterio-sclerosis Barr thinks that a low proteid diet is valuable and milk is in itself somewhat objectionable; as to drugs he recommends thyroid extract, the iodides and benzoates, and saline purgatives. On the other hand, M. Fränkel [2] thinks that iodine is useless in this disease, but finds considerable benefit from Trunecek's serum. When given hypodermically the process is tedious and painful, but antisclerosin, or the dried salts contained in it, can equally well be given by the mouth. The remedy may do good even in advanced cases, but it is still more valuable when given as a prophylactic as soon as we notice a high-tension pulse, slight dyspnœa, præcordial oppression, visual troubles, or the early signs of gout.

**Leucocytosis after Digestion.**—It appears that the absence of a normal increase of white cells after digestion is a strong, though not absolute, indication of gastric or hepatic cancer. The difficulties of diagnosing this condition are so great that any additional evidence is valuable. Goodall and others made observations on dogs and found the maximum rise four hours after food. The increase was chiefly in the lymphocytes and sometimes was shown also in the polymorphonuclear cells. It was also proved that the increase of cells was not due to increased action in the intestinal walls, but chiefly, if not entirely, to the bone-marrow. Other writers find that the maximum rise occurs in human beings two hours after food and is generally absent in gastric and hepatic cancer. In cases of gastric disease other than cancer, in tuberculosis, nephritis, diabetes, and other diseases, it is present except in the terminal stage, when gastric functions are completely abolished. Thus in ordinary cases its absence is strongly presumptive of malignant disease.

[1] Brit. Med. Jour., Jan. 20. [2] Amer. Jour. Med. Sci., Jan.

